# Design and Synthesis of Some Novel Fluorobenzimidazoles Substituted with Structural Motifs Present in Physiologically Active Natural Products for Antitubercular Activity

**Published:** 2017

**Authors:** Bangalore Nandha, Laxmivenkatesh Gurachar Nargund, Shachindra Laxmivenkatesh Nargund, Kishore Bhat

**Affiliations:** a *Department of Pharmaceutical Chemistry, Vivekananda College of Pharmacy, Rajiv Gandhi University of Health Sciences, Bangalore-560055, Karnataka, India.*; b *Department of Pharmaceutical Chemistry, Nargund College of Pharmacy, Rajiv Gandhi University of Health Sciences, Bangalore-560085, Karnataka, India. *; c *Department of Molecular Biology and Immunology, Maratha Mandalʹs NGH Institute of Dental Sciences & Research Centre, Belgaum-590010, Karnataka, India.*

**Keywords:** Fluorobenzimidazole, Antitubercular activity, *Mycobacterium tuberculosis*, H37Rv strain, Natural products

## Abstract

Keeping in view the drawbacks associated with research on anti-TB drugs based on plant extracts and the non-availability of fluorinated natural products with antitubercular activity has prompted us to make an effort towards the synthesis and characterization of a novel series of fifteen substituted fluorobenzimidazoles. The newly synthesized compounds were characterized by I.R, ^1^H-NMR, ^13^C-NMR, Mass, and elemental analysis. The synthesized compounds 4(a-f) and 5(b-j) have been evaluated for their *in*-*vitro* antimycobacterial activity against H37Rv strain (ATCC 27294) by MABA method. Incorporation of methylenedioxyphenyl moiety at 2- and 6-position of the benzimidazole ring furnished compounds 4d and 5i with antitubercular activity comparable or more potent than the naturally occurring compounds with reported antitubercular activity. Among the fifteen tested compounds, 4d and 5i emerged as promising hits characterized by MIC lower than that determined for sesamin against the pathogenic H37Rv strain. Antitubercular activity results indicate that these compounds may be suitable for further lead optimization. The cytotoxic effect of these active compounds on THP-1 cell line was assessed by MTT assay and the results suggest that these two molecules are potential candidates for further development as antitubercular agents.

## Introduction

Discovery of novel molecular scaffolds is a necessity to achieve effective control over multi-drug resistant strains of *Mycobacterium tuberculosis*. In light of this need, natural product research is one of the evolving strategies in identifying molecules with better anti-TB potential and safety profile than the currently available front line drugs (1-3). Low compound availability with relative structural complexity, and the cost involved in isolation of active principles from natural source are some of the practical difficulties associated with natural product research. Despite the abundance of fluorinated drugs in the market for the treatment of tuberculosis, there are no reports of fluorinated natural products with antitubercular activity. Therefore, incorporation of structural motifs present in natural products with potent antitubercular activity in fluorinated heterocyclic moieties of pharmaceutical interest is an important synthetic strategy in the design of potent anti-tubercular agents, and a better alternative to overcome the challenges with TB drug discovery based on natural products. Benzimidazole derivatives constitute an important class of therapeutic agents in medicinal chemistry and have been reported to have various biological activities, including antifungal (4), anticonvulsant (5), anticancer (6), anti-inflammatory (7), antioxidant (8), antihypertensive (9), antimicrobial (10), antiallergic (11), antiviral (12), antiprotozoal (13) and antihyperglycemic (14).

**Figure 1:**
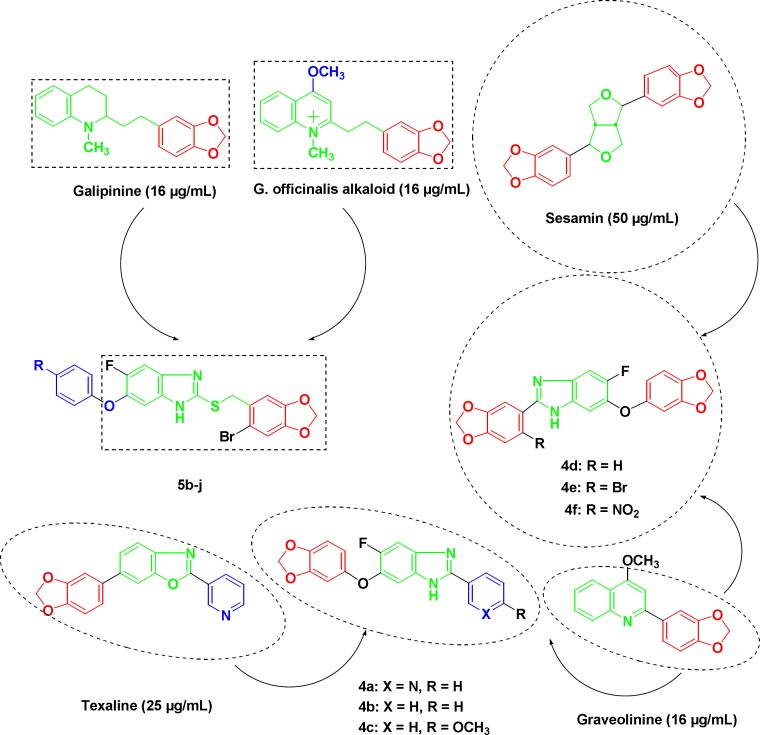
Structures of anti-tubercular natural products reported in literature and synthesized substituted fluorobenzimidazoles (4a-f and 5b-j).

The activity of synthetic pharmaceuticals, albendazole, and thiabendazole against *Mycobacterium tuberculosis *led to the identification of benzimidazole ring system and related compounds as small molecule inhibitors of bacterial cell division and were reported for their powerful antitubercular activity against H37Rv strain (15-18). SAR studies on the substituted benzimidazoles identified compounds with MIC values in the range of 0.63-12.5 µg/mL against the pathogenic bacilli (19-22). Substituted benzylsulfanyl benzimidazoles were synthesized and studied for their antitubercular activity towards MDR strains of *Mycobacterium tuberculosis* (23). Cinnamic acids and cinnamaldehydes have good potentials as anti-TB agents (24-27). Piperonylic acid is a natural molecule bearing a methylenedioxy function that closely mimics the structure of trans-cinnamic acid (28). Cinnamic benzimidazole derivatives were reported to exhibit good anti-TB activities on the H37Rv strain (29). Galipinine, sesamin, texaline and graveolinine are some of the methylenedioxyphenyl containing natural compounds having an inhibitory effect on *Mycobacterium tuberculosis* H37Rv strain (30-34). Antimycobacterial activity of these natural products is attributed to the presence of methylenedioxyphenyl ring as a key structural element in different heterocyclic scaffolds (Figure 1). These observations encouraged us to explore the different positions of the benzimidazole scaffold by the introduction of methylenedioxyphenyl moiety in an attempt to design and synthesize new anti-TB agents, and also incorporating fluorine atom in the molecule keeping in view the promising activity of fluorinated medicinals against *Mycobacterium tuberculosis*.

## Experimental

All reagents and solvents were purchased from commercial sources and used as received. IR spectra were recorded on a shimadzu-5400 FT-IR spectrometer with KBr discs and mass spectra were recorded on a LCMS 3200 triple quad (Q Trap) and LCMS 3000 API SCIEX mass spectrometer by electron spray ionization. Proton and ^13^C-NMR were run on a Bruker Avance-400 MHZ spectrometer (solutions in DMSO-d_6 _or CDCl_3_), with chemical shift values reported in *δ*, parts per million, relative to the internal standard. Thin layer chromatographic analyses were performed on 0.2 mm silica gel 60 F_254_ precoated (E-Merck) plates to monitor the reactions. The uncorrected melting points were determined in open glass capillaries. Elemental analyses were performed on Perkin-Elmer 2400 CHN elemental analyzer and the found values were within ± 0.4% of the theoretical values. Separations by chromatography on silica column were carried out by using silica gel (100-200 mesh) with ethyl acetate, hexane and petroleum ether as eluents of analytical grade.

*5-chloro-4-fluoro-2-nitroaniline (1)*

Yield (85%); mp 143-145 °C; IR (KBr) cm^-1^: 3493, 3319 (NH_2_ str), 3050 (Ar CH str), 1639, 1593, 1570 (C=C ring str), 1502 (NO_2_ str), 1242 (Ar C-N str), 1074 (C-F str), 1004 (C-Cl str). ^1^H-NMR (400 MHZ, CDCl_3_): *δ* 6.00 (br.s, 2H, NH_2_), 6.90-6.91 (d, 1H, H-3, *J* = 4.0 HZ, ArH), 7.91-7.94 (d, 1H, H-6, *J* = 12.0 HZ, ArH).^ 19^F-decoupled ^1^H-NMR (CDCl_3_): *δ* 4.75-6.20 (br.s, 2H, NH_2_), 6.92 (s, 1H, H-3, ArH), 7.93 (s, 1H, H-6, ArH).

*General procedure for the synthesis of 4-fluoro-5-(substituted)-2-nitroanilines (2 and 3)*

*A suspension of 5-chloro-4-fluoro-2-*nitroaniline** (**10 mmol), appropriate phenols (10 mmol), K_2_CO_3_ (20 mmol), and DMF (20 mL) was stirred at 90 °C for 15-18 h. Monitored the progress of the reaction by TLC. After the completion of the reaction, the reaction mixture was cooled at room temperature and poured into 200 mL of water followed by extraction with ethyl acetate (3×50 mL). The combined organic layer was washed with water and dried over anhydrous sodium sulphate. After filtration, the solvent was removed under reduced pressure to afford the nitro compounds. The crude yellow solid was used for the next step without further purification.

*5-(benzo[d][1, 3]dioxol-5-yloxy)-4-fluoro-2-nitrobenzenamine (3)*

Yield (81%); mp 206-208 °C; IR (KBr) cm^-1^: 3466, 3344 (NH_2_ str), 3186, 3058 (Ar CH str), 2906 (-OCH_2_O- str), 1644 (C=N str), 1508 (Ar NO_2_ str), 1599, 1487 (C=C ring str), 1360 (Ar C-N str), 1249 (Ar C-O-C str), 1176 ( C-F str).^ 1^H-NMR (DMSO-d_6_): *δ* 6.09 (s, 2H, -OCH_2_O-), 6.34-6.36 (d, 1H, *J* = 8.0 HZ, ArH), 6.67-6.70 (d, 1H, *J* = 12.0 HZ, ArH), 6.94 (s, 1H, ArH), 6.98-7.01 (d, 1H, *J* = 12.0 HZ, ArH), 7.40 (br.s, 2H, NH_2_), 7.86-7.89 (d, 1H, *J* = 12.0 HZ, ArH).

*General procedure for the synthesis of 4-(substituted)-5-fluorobenzene-1,2-diamine (2a and 3a)*

To a solution of nitro compound (1 mmol) in ethanol (10 mL) was added SnCl_2_.2H_2_O (7 mmol). The reaction mixture was stirred under reflux at 75 °C for 5-7 h and monitored by TLC for completion of the reaction. After completion, sufficient 10% NaOH solution was added until the solution was just alkaline to litmus and extracted with ethyl acetate (3×50 mL). The combined organic layer was washed with brine (50 mL), water and dried over anhydrous sodium sulphate. The solvent was removed under reduced pressure to afford the corresponding 4-(substituted)-5-fluorobenzene-1,2-diamine. Because of the instability of the diamine, the reduced compound was used for the next step without further purification.

*General procedure for the synthesis of 5-fluoro-6-(substituted)-1H-benzo[d]imidazole-2-thiol*

*(3b-j)*

Carbon disulphide (10 mmol) was added to a stirred suspension of 4-(substituted)-5-fluorobenzene-1,2-diamine (2a) (5 mmol) and potassium hydroxide (10 mmol) in 25 mL of rectified spirit. The reaction mixture was stirred under reflux at 75 °C for 5-7 h and monitored by TLC for completion of the reaction. After completion, the reaction mixture was gradually cooled to room temperature and was quenched with water followed by treatment with sufficient 30% acetic acid solution to pH 5. The separated solid was filtered, washed with water and suck dried to yield the crude substituted-2-mercaptobenzimidazoles. The crude product was purified by recrystallization from aqueous ethanol to yield pure (3b-j).

*6-chloro-5-fluoro-1H-benzo[d]imidazole-2-thiol (3b)*

Yield (71%); mp 291-294 °C; IR (KBr) cm^-1^: 3400 (NH str), 3117, 3070 (Ar CH str), 1625 (C=N str), 1520, 1500, 1465 (C=C ring str), 1155 (Ar C-F str), 1050 (Ar C-Cl str). ^1^H-NMR (DMSO-d_6_): *δ* 7.16-7.18 (d, 1H, *J* = 8.0 HZ, ArH), 7.24-7.26 (d, 1H, *J* = 8.0 HZ, ArH), 12.69 (s, 1H, NH or SH), 12.76 (s, 1H, NH or SH). MS (ESI) m/z: 201 (M-1).

*5-fluoro-6-(naphthalen-2-yl oxy)-1H-benzo[d]imidazole-2-thiol (3c)*

Yield (79%); mp 246-248 °C; IR (KBr) cm^-1^: 3406 (NH str), 3126, 3064 (Ar CH str), 1626 (C=N str), 1599, 1579, 1480 (C=C ring str), 1232 (Ar C-O-C str), 1163 (Ar C-F str). ^1^H-NMR (DMSO-d_6_): *δ* 6.78-6.80 (d, 1H, *J* = 8.0 HZ, ArH), 6.89-6.91 (d, 1H, *J* = 8.0 HZ, ArH), 7.20-7.22 (d, 1H, *J* = 8.0 HZ, ArH), 7.37-7.39, 7.39-7.41 (t, 1H, *J* = 8.0 HZ, *J* = 8.0 HZ, ArH), 7.56-7.62 (m, 2H, ArH), 7.66-7.69 (d, 1H, *J* = 12.0 HZ, ArH), 7.96-7.98 (m, 1H, ArH), 8.21 (m, 1H, ArH), 12.52 (s, 1H, NH or SH), 12.67 (s, 1H, NH or SH). MS (ESI) m/z: 309.1 (M-1). 

*5-fluoro-6-(4-phenylphenoxy)-1H-benzo[d]imidazole-2-thiol (3d)*

Yield (59%); mp 152-155 °C; IR (KBr) cm^-1^: 3400 (NH str), 3080, 3005 (Ar CH str), 1626 (C=N str), 1605, 1518, 1475 (C=C ring str), 1367 (Ar C-N str), 1230 (Ar C-O-C str), 1172 (Ar C-F str). ^1^H-NMR (DMSO-d_6_): *δ* 6.94-6.96 (d, 1H, *J* = 8.0 HZ, ArH), 7.02-7.04 (d, 2H, *J* = 8.0 HZ, ArH), 7.17-7.19 (d, 1H, *J* = 8.0 HZ, ArH), 7.31-7.34 (t, 1H, ArH), 7.41-7.45 (m, 2H, ArH), 7.60-7.64 (m, 4H, ArH), 12.57 (s, 1H, NH or SH), 12.67 (s, 1H, NH or SH). MS (ESI) m/z: 337.2 (M+1).

*5-fluoro-6-(p-tolyloxy)-1H-benzo[d]imidazole-2-thiol (3e)*

Yield (69%); mp 282-285 °C; IR (KBr) cm^-1^: 3394 (NH str), 3124, 3082 (Ar CH str), 2989, 2955 (aliphatic CH_3_ str), 1626 (C=N str), 1606, 1508, 1476 (C=C ring str), 1217 (Ar C-O-C str), 1159 (Ar C-F str). ^1^H-NMR (DMSO-d_6_): *δ* 2.25 (s, 3H, CH_3_), 6.80-6.81 (d, 1H, *J* = 4.0 HZ, ArH), 6.83-6.87 (d, 2H, ArH), 7.12-7.14 (d, 2H, *J* = 8.0 HZ, ArH), 7.15-7.16 (d, 1H, *J* = 4.0 HZ, ArH), 12.50 (s, 1H, NH or SH), 12.65 (s, 1H, NH or SH). MS (ESI) m/z: 273.1 (M-1).

*6-(4-tert-butylphenoxy)-5-fluoro-1H-benzo[d]imidazole-2-thiol (3f)*

Yield (59%); mp 152-155 °C; IR (KBr) cm^-1^: 3421 (NH str), 3120, 3080 (Ar CH str), 2970 (CH_3_ str), 1630 (C=N str), 1545, 1512, 1480 (C=C ring str), 1362 (Ar C-N str), 1230 (Ar C-O-C str), 1180 (Ar C-F str). ^1^H-NMR (DMSO-d_6_): *δ* 1.25 (s, 9H, (CH_3_)_3_), 6.84-6.88 (m, 3H, ArH), 7.13-7.15 (d, 1H, *J* = 8.0 HZ, ArH), 7.34-7.36 (d, 2H, *J* = 8.0 HZ, ArH), 12.50 (br.d, 2H, NH and SH). MS (ESI) m/z: 317.3 (M+1).

*6-(4-chloro phenoxy)-5-fluoro-1H-benzo[d]imidazole-2-thiol (3g)*

Yield (77%); mp 237-239 °C; IR (KBr) cm^-1^: 3490 (NH str), 3130, 3082 (Ar CH str), 1650 (C=N str), 1593, 1471 (C=C ring str), 1226 (Ar C-O-C str), 1170 (Ar C-F str), 1101 (Ar C-Cl str). ^1^H-NMR (DMSO-d_6_): *δ* 6.93-6.95 (d, 1H, *J* = 8.0 HZ, ArH), 6.97-6.99 (d, 2H, *J* = 8.0 HZ, ArH), 7.16-7.18 (d, 1H, *J* = 8.0 HZ, ArH), 7.37-7.40 (d, 2H, *J* = 12.0 HZ, ArH), 12.59 (s, 1H, NH or SH), 12.68 (s, 1H, NH or SH). MS (ESI) m/z: 293.0 (M-1).

*5-fluoro-6-(4-fluorophenoxy)-1H-benzo[d]imidazole-2-thiol (3h)*

Yield (85%); mp 257-259 °C; IR (KBr) cm^-1^: 3495 (NH str), 3093 (Ar CH str), 1630 (C=N str), 1595, 1475 (C=C ring str), 1229 (Ar C-O-C str), 1173 (Ar C-F str). ^1^H-NMR (DMSO-d_6_): *δ* 6.85-6.87 (d, 1H, *J* = 8.0 HZ, ArH), 6.98-7.02 (m, 2H, ArH), 7.14-7.20 (m, 3H, ArH), 12.59 (br.s, 2H, NH and SH). MS (ESI) m/z: 277.2 (M-1).

*6-(benzo[d] [1,3]dioxol-5-yloxy)-5-fluoro-1H-benzo[d]imidazole-2-thiol (3i)*

Yield (82%); mp 269-271 °C; IR (KBr) cm^-1^ : 3559 (NH str), 3052 (Ar CH str), 2917 (-OCH_2_O- str), 2551 (SH str), 1628 (C=N str), 1603, 1478 (C=C ring str), 1365 (Ar C-N str), 1246 (Ar C-O-C str), 1176 ( C-F str). ^1^H-NMR (DMSO-d_6_): *δ* 6.01 (s, 2H, -OCH_2_O-), 6.39-6.42 (d, 1H, *J* = 12.0 HZ, ArH), 6.69-7.71 (d, 1H, *J* = 8.0 HZ, ArH), 6.77-7.79 (d, 1H, *J* = 8.0 HZ, ArH), 6.84-6.86 (d, 1H, *J* = 8.0 HZ, ArH), 7.11-7.14 (d, 1H, *J* = 12.0 HZ, ArH), 12.48 (s, 1H, SH or NH), 12.61 (s, 1H, SH or NH). MS (ESI) m/z: 303.0 (M-1).

*6-(4-(1H-imidazol-1-yl) phenoxy)-5-fluoro-1H-benzo[d]imidazole-2-thiol (3j)*

Yield (73%); mp 275-277 °C; IR (KBr) cm^-1^: 3474 (NH str), 3058 (Ar CH str), 2575 (SH str), 1628 (C=N str), 1517, 1478 (C=C ring str), 1369 (Ar C-N str), 1234 (Ar-O-Ar, C-O-C str), 1176 (C-F str).^ 1^H-NMR (DMSO): δ 6.94-6.96 (d, 1H, J = 8.0 HZ, ArH), 7.07-7.10 (m, 3H, ArH), 7.16-7.19 (d, 1H, J = 12.0 HZ, ArH), 7.58-7.66 (m, 3H, ArH), 8.15 (s, 1H, imidazole), 12.60 (s, 1H, SH or NH), 12.66 (s, 1H, SH or NH). MS (ESI) m/z: 327.1 (M+1).

*General procedure for the synthesis of6-(benzo[d][1,3]dioxol-5-yloxy)-5-fluoro-2-(substitutedphenyl)-1H-benzo[d]imidazole(4a-f)*

A mixture of 4-(benzo[d] [1, 3] dioxol-5-yloxy)-5-fluorobenzene-1, 2-diamine (3a) (10 mmol), appropriate aldehyde (10 mmol) and sodium metabisulfite (11 mmol) in dry DMF (10 mL) was heated at 120 °C with stirring under nitrogen atmosphere for 18 h. Once thin-layer chromatography showed the absence of starting materials with appearance of a new spot, the reaction mixture was cooled to room temperature, poured into water (100 mL) and extracted with ethyl acetate. The ethyl acetate layer was dried over anhydrous Na_2_SO_4 _and filtered. The organic extracts were concentrated in vacuo and the crude product was purified by recrystallization from ethyl alcohol to afford (4a-f).

*6-(benzo[d] [1,3]dioxol-5-yloxy)-5-fluoro-2-(pyridin-3-yl)-1H-benzo[d]imidazole (4a)*

Yield (59%); mp 167-169 °C; IR (KBr) cm^-1^: 3468 (NH str), 3105, 3040 (Ar CH str), 2910 (-OCH_2_O- str), 1630 (C=N str), 1605, 1470 (C=C ring str), 1355 (Ar C-N str), 1241 (Ar C-O-C str), 1175 ( C-F str).^ 1^H-NMR (CDCl_3_): *δ* 5.97 (s, 2H, -OCH_2_O-), 7.18-7.36 (m, 7H, ArH), 7.73 (s, 1H, ArH), 8.32-8.34 (t, 1H, ArH), 11.01 (s, 1H, NH). MS (ESI) m/z: 350.2 (M+1). Anal. Calcd for C_19_H_12_FN_3_O_3_: C, 65.33; H, 3.46; N, 12.02. Found: C, 65.21; H, 3.37; N, 11.91.

*6-(benzo[d][1,3]dioxol-5-yloxy)-5-fluoro-2-phenyl-1H-benzo[d]imidazole (4b)*

Yield (60%); mp 185-187 °C; IR (KBr) cm^-1^: 3468 (NH str), 3101, 3055 (Ar CH str), 2923 (-OCH_2_O- str), 1634 (C=N str), 1610, 1482 (C=C ring str), 1359 (Ar C-N str), 1247 (Ar C-O-C str), 1180 ( C-F str).^ 1^H-NMR (DMSO-d_6_): *δ* 6.02 (s, 2H, -OCH_2_O-), 6.36-6.42 (m, 1H, ArH), 6.68-6.74 (m, 1H, ArH), 6.83-6.87 (m, 1H, ArH), 7.20 (s, 1H, ArH), 7.40-7.70 (m, 4H, ArH), 8.10-8.13 (d, 2H,* J* = 12.0 HZ, ArH), 12.94 (br.s, 1H, NH).^ 13^C-NMR (DMSO-d_6_): *δ *99.99, 101.45, 103.13, 105.76, 105.96, 108.18, 108.25, 109.01, 111.16, 126.29, 128.61, 128.77, 128.96, 129.74, 129.96, 131.05, 131.53, 143.05, 148.14, 152.38. MS (ESI) m/z: 348.2 (M-1). Anal. Calcd for C_20_H_13_FN_2_O_3_: C, 68.96; H, 3.76; N, 8.03. Found: C, 68.82; H, 3.60; N, 7.93.

*6-(benzo[d][1,3]dioxol-5-yloxy)-5-fluoro-2-(4-methoxyphenyl)-1H-benzo[d]imidazole (4c)*

Yield (63%); mp 235-237 °C; IR (KBr) cm^-1^: 3480 (NH str), 3168, 3045 (Ar CH str), 2921 (-OCH_2_O- str), 2857 (Aliphatic CH_3_ str), 1673 (C=N str), 1610, 1481 (C=C ring str), 1359 (Ar C-N str), 1255 (Ar-O-Ar, C-O-C str), 1179 ( C-F str), 1035 (Ar-O-R, C-O-C str).^ 1^H-NMR (DMSO-d_6_): *δ* 3.82 (s, 3H, OCH_3_), 5.99 (s, 2H, -OCH_2_O-), 6.32-6.39 (m, 1H, ArH), 6.67-6.77 (m, 1H, ArH), 6.84 (br.s, 1H, ArH), 7.05-7.12 (m, 1H, ArH), 7.37-7.40 (d, 1H, *J* = 12.0 HZ, ArH), 7.43-7.45 (d, 1H, *J* = 8.0 HZ, ArH), 7.52-7.59 (m, 1H, ArH), 7.67-7.71 (m, 1H, ArH), 8.04-8.06 (d, 1H,* J* = 8.0 HZ, ArH), 12.78 (br.s, 1H, NH).^ 13^C-NMR (DMSO-d_6_): *δ *55.82, 99.89, 100.35, 101.98, 103.61, 108.72, 109.32, 114.66, 114.73, 114.90, 122.81, 128.42, 129.12, 131.53, 132.04, 140.35, 143.50, 148.64, 153.42. MS (ESI) m/z: 378.2 (M-1). Anal. Calcd for C_21_H_15_FN_2_O_4_: C, 66.66; H, 3.99; N, 7.40. Found: C, 66.58; H, 3.90; N, 7.31.

*2-(benzo[d][1,3]dioxol-5-yl)-6-(benzo[d][1,3]dioxol-5-yloxy)-5-fluoro-1H-benzo[d]imidazole (4d)*

Yield (71%); mp 217-219 °C; IR (KBr) cm^-1^: 3431 (NH str), 3065, 3058 (Ar CH str), 2903 (-OCH_2_O- str), 1622 (C=N str), 1510, 1481 (C=C ring str), 1341 (Ar C-N str), 1242 (Ar C-O-C str), 1178 ( C-F str).^ 1^H-NMR (DMSO-d_6_): *δ* 6.00 (s, 2H, -OCH_2_O-), 6.11 (s, 2H, -OCH_2_O-), 6.34-6.42 (m, 1H, ArH), 6.67-6.73 (m, 1H, ArH), 6.82-6.84 (d, 1H, *J* = 8.0 HZ, ArH), 7.07-7.09 (d, 1H, *J* = 8.0 HZ, ArH), 7.17 (s, 1H, ArH), 7.46 (s, 1H, ArH), 7.57-7.67 (m, 2H, ArH), 12.90 (br.s, 1H, NH). MS (ESI) m/z: 392.0 (M-1). Anal. Calcd for C_21_H_13_FN_2_O_5_: C, 64.28; H, 3.34; N, 7.13. Found: C, 64.08; H, 3.16; N, 7.03.

*6-(benzo[d][1,3]dioxol-5-yloxy)-2-(6-bromobenzo[d][1,3]dioxol-5-yl)-5-fluoro-1H-benzo[d]imidazole (4e)*

Yield (66%); mp 197-199 °C; IR (KBr) cm^-1^: 3455 (NH str), 3102, 3040 (Ar CH str), 2904 (-OCH_2_O- str), 1683 (C=N str), 1610, 1481 (C=C ring str), 1347 (Ar C-N str), 1242 (Ar C-O-C str), 1178 ( C-F str), 674 ( C-Br).^ 1^H-NMR (DMSO-d_6_): *δ* 5.96 (s, 2H, -OCH_2_O-), 6.07 (s, 2H, -OCH_2_O-), 6.46 (br.s, 1H, ArH), 6.60 (s, 1H, ArH), 6.70-6.75 (m, 2H, ArH), 7.05-7.13 (m, 2H, ArH), 7.75 (s, 1H, ArH), 10.01 (br.s, 1H, NH).^ 13^C-NMR (CDCl_3_): *δ *100.42, 100.65, 101.47, 101.55, 102.37, 102.51, 102.73, 106.60, 106.81, 108.13, 108.20, 109.62, 109.98, 111.32, 111.64, 113.56, 123.69, 128.80, 130.86, 148.10, 149.91. MS (ESI) m/z: 473.1 (M+2). Anal. Calcd for C_21_H_12_BrFN_2_O_5_: C, 53.52; H, 2.56; N, 5.94. Found: C, 53.35; H, 2.43; N, 5.81.

*6-(benzo[d][1,3]dioxol-5-yloxy)-5-fluoro-2-(6-nitrobenzo[d][1,3]dioxol-5-yl)-1H-benzo[d]imidazole (4f)*

Yield (65%); mp 222-225 °C; IR (KBr) cm^-1^: 3375 (NH str), 3048 (Ar CH str), 2964, 2884 (-OCH_2_O- str), 1634 (C=N str), 1612, 1482 (C=C ring str), 1500 (Ar NO_2_ str), 1369 (Ar C-N str), 1224 (Ar C-O-C str), 1179 ( C-F str).^ 1^H-NMR (DMSO-d_6_): *δ* 5.77 (s, 1H, ArH), 5.92 (s, 2H, -OCH_2_O-), 5.94-5.95 (d, 1H, ArH), 5.96 (s, 2H, -OCH_2_O-), 6.80-6.82 (d, 1H, *J* = 8.0 HZ, ArH), 6.89-6.91 (d, 1H, *J* = 8.0 HZ, ArH), 7.00 (d, 1H, ArH), 7.07 (s, 2H, ArH), 12.85 (br.s, 1H, NH). MS (ESI) m/z: 437.2 (M+1). Anal. Calcd for C_21_H_12_FN_3_O_7_: C, 57.67; H, 2.76; N, 9.60. Found: C, 57.57; H, 2.65; N, 9.52.

*General procedure for the synthesis of2-((6-bromobenzo[d][1,3]dioxol-5-yl)methylthio)-5-fluoro-6-(substituted)-1H-benzo[d]imidazole (5b-j)*

Powdered potassium carbonate (4 mmol) was added to a stirred mixture of (3b-j) (2 mmol) and 5-Bromo-6-bromomethyl-1, 3-benzodioxole (4) (2 mmol) in dry DMF under nitrogen atmosphere. The resultant mixture was stirred at room temperature over different periods till the completion of the reaction, confirmed by TLC. The reaction mixture was quenched with water (100 mL), extracted with ethyl acetate and the organic layer was separated. The combined organic layer was washed with water followed by brine and finally dried over anhydrous Na_2_SO_4_. Solvent was removed under reduced pressure and the crude product was purified by column chromatography on silica gel (100-200 mesh) eluting with 2-20% ethyl acetate-hexane to yield pure (5b-j).

*2-((6-bromobenzo[d][1,3]dioxol-5-yl)methylthio)-6-chloro-5-fluoro-1H-benzo[d]imidazole (5b)*

Yield (55%); mp 210-212 °C; IR (KBr) cm^-1^: 3585 (NH str), 3052 (Ar CH str), 2966 (-OCH_2_O- str), 2881 (Aliphatic CH_2_ str), 1646 (C=N str), 1506, 1483 (C=C ring str), 1344 (Ar C-N str), 1242 (Ar-O-Ar, C-O-C str), 1037 (C-F str), 968 ( C-Cl str), 672 (C-Br str).^ 1^H-NMR (DMSO-d_6_): *δ* 4.54 (s, 2H, -SCH_2_-), 6.03 (s, 2H, -OCH_2_O-), 7.18 (s, 1H, ArH), 7.23 (s, 1H, ArH), 7.50-7.52 (d, 1H, *J* = 8.0 HZ, ArH), 7.64-7.66 (d, 1H, *J* = 8.0 HZ, ArH), 12.50 (br.s, 1H, NH). MS (ESI) m/z: 417.2 (M+2). Anal. Calcd for C_15_H_9_BrClFN_2_O_2_S: C, 43.34; H, 2.18; N, 6.73. Found: C, 43.16; H, 2.05; N, 6.64.

*2-((6-bromobenzo[d][1,3]dioxol-5-yl)methylthio)-5-fluoro-6-(naphthalene-2-yloxy)-1H-benzo[d]imidazole (5c)*

Yield (59%); mp 196-198 °C; IR (KBr) cm^-1^: 3620 (NH str), 3040 (Ar CH str), 2964 (-OCH_2_O- str), 2872 (Aliphatic CH_2_ str), 1628 (C=N str), 1573, 1500, 1477 (C=C ring str), 1353 (Ar C-N str), 1232 (Ar-O-Ar, C-O-C str), 1176 (C-F str), 672 (C-Br str).^ 1^H-NMR (CDCl_3_): *δ* 4.49 (s, 2H, -SCH_2_-), 5.90 (s, 2H, -OCH_2_O-), 6.63-6.65 (d, 1H, *J* = 8.0 HZ, ArH), 6.80-7.02 (m, 2H, ArH), 7.13-7.15 (d, 1H, *J* = 8.0 HZ, ArH), 7.22 (s, 1H, ArH), 7.24-7.30 (m, 2H, ArH), 7.46-7.49 (d, 2H, *J* = 12.0 HZ, ArH), 7.79-7.81 (s, 1H, *J* = 12.0 HZ, ArH), 8.27 (s, 1H, ArH), 13.07 (br.s, 1H, NH).^ 13^C-NMR (CDCl_3_): *δ* 36.18, 101.42, 107.80, 108.98, 109.43, 110.24, 111.97, 114.37, 121.18, 121.82, 125.22, 125.33, 126.16, 126.39, 127.10, 129.17, 130.55, 134.07, 138.45, 146.78, 147.49, 149.11, 150.74, 153.65. MS (ESI) m/z: 525.1 (M+2). Anal. Calcd for C_25_H_16_BrFN_2_O_3_S: C, 57.37; H, 3.08; N, 5.34. Found: C, 57.17; H, 2.99; N, 5.25.

*2-((6-bromobenzo[d][1,3]dioxol-5-yl)methylthio)-5-fluoro-6-(4-phenylphenoxy)-1H-benzo[d]imidazole (5d)*

Yield (57%); mp 188-190 °C; IR (KBr) cm^-1^: 3480 (NH str), 3034 (Ar CH str), 2981 (-OCH_2_O- str), 2887 (Aliphatic CH_2_ str), 1648 (C=N str), 1606, 1500, 1476 (C=C ring str), 1358 (Ar C-N str), 1238 (Ar-O-Ar, C-O-C str), 1038 (C-F str), 695 (C-Br str). ^1^H-NMR (CDCl_3_): *δ* 4.73 (s, 2H, -SCH_2_-), 5.93 (s, 2H, -OCH_2_O-), 6.75 (s, 1H, ArH), 6.86 (s, 1H, ArH), 6.86-6.91 (m, 3H, ArH), 6.92-7.06 (m, 6H, ArH), 7.33-7.35 (d, 1H, *J* = 8.0 HZ, ArH), 7.59-7.61 (d, 1H, *J* = 8.0 HZ, ArH), 15.10 (br.s, 1H, NH).^ 13^C-NMR (CDCl_3_): *δ* 39.32, 101.62, 101.87, 102.11, 105.43, 110.91, 112.95, 115.65, 117.72, 126.17, 126.74, 127.07, 128.23, 128.49, 128.64, 137.06, 140.02, 147.65, 148.90, 149.89, 156.13. MS (ESI) m/z: 551.1 (M+2). Anal. Calcd for C_27_H_18_BrFN_2_O_3_S: C, 59.02; H, 3.30; N, 5.09. Found: C, 58.87; H, 3.19; N, 5.04.

*2-((6-bromobenzo[d][1,3]dioxol-5-yl)methylthio)-5-fluoro-6-(p-tolyloxy)-1H-benzo[d]imidazole (5e)*

Yield (53%); mp 205-207 °C; IR (KBr) cm^-1^: 3370 (NH str), 3065 (Ar CH str), 2957 (-OCH_2_O- str), 2898 (Aliphatic CH_3_ str), 1625 (C=N str), 1600, 1581, 1500, 1481 (C=C ring str), 1363 (Ar C-N str), 1239 (Ar-O-Ar, C-O-C str), 1163 (C-F str), 569 (C-Br str).^ 1^H-NMR (DMSO-d_6_): *δ* 2.23 (s, 3H, CH_3_), 4.56 (s, 2H, -SCH_2_-), 6.00 (s, 2H, -OCH_2_O-), 6.81 (s, 2H, ArH), 6.95-7.07 (m, 2H, ArH), 7.20-7.22 (d, 1H, *J* = 8.0 HZ, ArH), 7.31-7.33 (d, 2H, *J* = 8.0 HZ, ArH), 7.88-7.89 (d, 1H, *J* = 4.0 HZ, ArH), 12.97 (br.s, 1H, NH).^ 13^C-NMR (DMSO-d_6_): *δ *20.13, 34.02, 101.50, 101.55, 106.38, 107.45, 107.50, 118.30, 123.27, 125.38, 126.63, 130.60, 131.11, 131.41, 131.73, 141.58, 142.17, 151.08, 155.05. MS (ESI) m/z: 489.2 (M+2). Anal. Calcd for C_22_H_16_BrFN_2_O_3_S: C, 54.22; H, 3.30; N, 5.74. Found: C, 54.04; H, 3.16; N, 5.70.

*2-((6-bromobenzo[d][1,3]dioxol-5-yl)methylthio)-6-(4-tert-butylphenoxy)-5-fluoro-1H-benzo[d]imidazole (5f)*

Yield (63%); mp 175-177 °C; IR (KBr) cm^-1^: 3517 (NH str), 3040 (Ar CH str), 2961 (-OCH_2_O- str), 2906 (Aliphatic CH_2_ str), 2870 (Aliphatic CH_3_ str), 1744 (C=N str), 1601, 1506, 1478 (C=C ring str), 1350 (Ar C-N str), 1240 (Ar-O-Ar, C-O-C str), 1170 (C-F str), 553 (C-Br str).^ 1^H-NMR (CDCl_3_): *δ* 1.26 (s, 3H, CH_3_), 1.32 (s, 6H, 2CH_3_), 4.58 (s, 2H, -SCH_2_-), 5.96 (s, 2H, -OCH_2_O-), 6.89-6.91 (d, 2H, *J* = 8.0 HZ, ArH), 6.97-7.02 (m, 4H, ArH), 7.18 (br.s, 1H, ArH), 7.31-7.34 (m, 1H, *J* = 12.0 HZ, ArH), 10.50 (br.s, 1H, NH). MS (ESI) m/z: 531.3 (M+2). Anal. Calcd for C_25_H_22_BrFN_2_O_3_S: C, 56.71; H, 4.18; N, 5.28. Found: C, 56.59; H, 4.08; N, 5.19.

*2-((6-bromobenzo[d][1,3]dioxol-5-yl)methylthio)-6-(4-chlorophenoxy)-5-fluoro-1H-benzo[d]imidazole (5g)*

Yield (60%); mp 162-164 °C; IR (KBr) cm^-1^: 3499 (NH str), 3040 (Ar CH str), 2978 (-OCH_2_O- str), 2887 (Aliphatic CH_2_ str), 1622 (C=N str), 1586, 1480 (C=C ring str), 1353 (Ar C-N str), 1242 (Ar-O-Ar, C-O-C str), 1037 (C-F str), 968 (C-Br str), 669 (C-Br str).^ 1^H-NMR (DMSO-d_6_): *δ* 4.56 (s, 2H, -SCH_2_-), 6.04 (s, 2H, -OCH_2_O-), 6.94-6.96 (d, 2H, *J* = 12.0 HZ, ArH), 7.19 (s, 1H, ArH), 7.23 (s, 1H, ArH), 7.34-7.39 (m, 3H, ArH), 7.52-7.55 (d, 1H, *J* = 12.0 HZ, ArH), 12.61 (br.s, 1H, NH).^ 13^C-NMR (DMSO-d_6_): *δ* 36.35, 101.87, 102.12, 103.27, 107.12, 109.19, 110.64, 112.19, 112.51, 114.65, 117.71, 126.43, 128.95, 129.66, 137.87, 138.01, 147.09, 147.98, 149.51, 151.13, 151.89, 156.89. MS (ESI) m/z: 508.3 (M+2). Anal. Calcd for C_21_H_13_BrClFN_2_O_3_S: C, 49.67; H, 2.58; N, 5.51. Found: C, 49.46; H, 2.43; N, 5.45.

*2-((6-bromobenzo[d][1,3]dioxol-5-yl)methylthio)-5-fluoro-6-(4-fluorophenoxy)-1H-*

*benzo [d]imidazole (5h)*

Yield (58%); mp 150-152 °C; IR (KBr) cm^-1^: 3444 (NH str), 3046 (Ar CH str), 2975 (-OCH_2_O- str), 2888 (Aliphatic CH_2_ str), 1630 (C=N str), 1597, 1500, 1475 (C=C ring str), 1347 (Ar C-N str), 1244 (Ar-O-Ar, C-O-C str), 1036 (C-F str), 672 (C-Br str).^ 1^H-NMR (CDCl_3_): *δ* 4.91 (s, 2H, -SCH_2_-), 5.93 (s, 2H, -OCH_2_O-), 6.99-7.01 (d, 1H, ArH), 7.28-7.30 (m, 1H, ArH), 7.38-7.41 (m, 2H, ArH), 7.51-7.61 (m, 4H, ArH), 12.50 (br.s, 1H, NH).^ 13^C-NMR (CDCl_3_): *δ* 38.05, 101.64, 101.76, 101.99, 109.11, 110.63, 112.85, 115.32, 116.34, 118.44, 128.60, 131.22, 141.26, 147.42, 148.29, 150.52, 153.70, 157.42. MS (ESI) m/z: 493.2 (M+2). Anal. Calcd for C_21_H_13_BrF_2_N_2_O_3_S: C, 51.33; H, 2.66; N, 5.69. Found: C, 51.16; H, 2.47; N, 5.62.

*6-(benzo[d][1,3]dioxol-5-yloxy)-2-((6-bromobenzo[d][1,3]dioxol-5-yl)methylthio)-5-fluoro-1H-benzo[d]imidazole (5i)*

Yield (65%); mp 222-224 °C; IR (KBr) cm^-1^: 3521 (NH str), 3028 (Ar CH str), 2982 (-OCH_2_O- str), 2895 (Aliphatic CH_2_ str), 1618 (C=N str), 1506, 1479 (C=C ring str), 1353 (Ar C-N str), 1246 (Ar-O-Ar, C-O-C str), 1170 (C-F str), 629 (C-Br str).^ 1^H-NMR (CDCl_3_): *δ* 4.88 (s, 2H, -SCH_2_-), 5.93 (s, 2H, -OCH_2_O-), 5.95 (s, 2H, -OCH_2_O-), 6.42-6.44 (d, 1H, *J* = 8.0 HZ, ArH), 6.71-7.73 (d, 1H, *J* = 8.0 HZ, ArH), 6.76 (s, 1H, ArH), 6.85 (s, 1H, ArH), 6.92-6.99 (m, 1H, ArH), 7.14 (s, 1H, ArH), 7.52-7.54 (d, 1H, *J* = 8.0 HZ, ArH), 10.14 (br.s, 1H, NH).^ 13^C-NMR (CDCl_3_): *δ* 36.31, 101.07, 101.68, 101.78, 102.13, 108.30, 110.17, 110.80, 110.97, 112.63, 112.79, 112.97, 126.30, 128.19, 138.00, 144.54, 147.86, 149.04, 150.94, 159.00. MS (ESI) m/z: 519.0 (M+2). Anal. Calcd for C_22_H_16_BrFN_2_O_3_S: C, 51.07; H, 2.72; N, 5.41. Found: C, 50.92; H, 2.63; N, 5.36.

*6-(4-(1H-imidazol-1-yl) phenoxy)-2-((6-bromobenzo[d][1,3]dioxol-5-yl)methylthio)-5-fluoro-1H-benzo[d]imidazole (5j)*

Yield (61%); mp 245-247 °C; IR (KBr) cm^-1^: 3523 (NH str), 3040 (Ar CH str), 2979 (-OCH_2_O- str), 2893 (Aliphatic CH_2_ str), 1610 (C=N str), 1511, 1477 (C=C ring str), 1353 (Ar C-N str), 1237 (Ar-O-Ar, C-O-C str), 1170 (C-F str), 534 (C-Br str).^ 1^H-NMR (DMSO-d_6_): *δ* 4.55 (s, 2H, -SCH_2_-), 6.04 (s, 2H, -OCH_2_O-), 7.00-7.06 (m, 3H, ArH), 7.19-7.24 (m, 3H, ArH), 7.42-7.45 (d, 1H, *J* = 12.0 HZ, ArH), 7.59 (br.s, 2H, *J* = 8.0 HZ, ArH), 7.73 (br.s, 1H, ArH), 8.24 (br.s, 1H, ArH), 12.80 (br.s, 1H, NH). MS (ESI) m/z: 541.2 (M+2). Anal. Calcd for C_24_H_16_BrFN_4_O_3_S: C, 53.44; H, 2.99; N, 10.38. Found: C, 53.26; H, 2.85; N, 10.29.

*Microbiology*

All the newly synthesized benzimidazole derivatives were screened for their *in-vitro *antitubercular activity against *M. tuberculosis* H37Rv strain (ATCC 27294), and *in-vitro *assay was performed for evaluation of cytotoxicity of the analogues 4d and 5h on THP-1 cell line by MTT assay.

*Antitubercular activity*

A facile and efficient visual Microplate Alamar Blue Assay (MABA) method was adopted for the screening of test compounds against *M. tuberculosis* H37Rv strain. Visual MABA is a promising alternative, not only for providing identical and rapid results but also in view of the good correlation between the MICs determined by BACTEC, fluorometric MABA and visual MABA methods (35). This colorimetric method involves the use of a thermally stable and nontoxic redox indicator.

**Figure 2.**
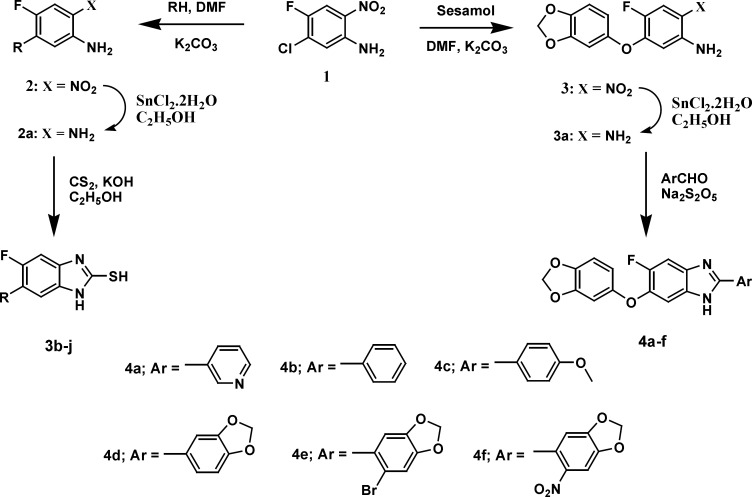
Synthesis of mercaptobenzimidazoles 3(b-j) and benzimidazole derivatives 4(a-f).

**Figure 3:**
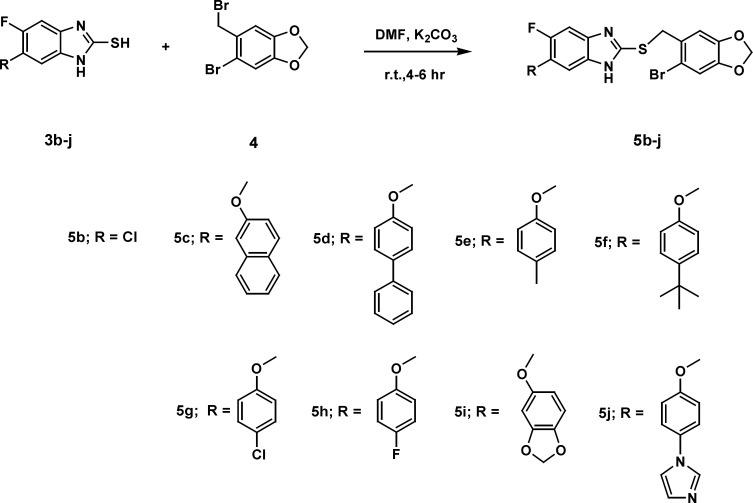
Synthesis of compounds 5(b-j).

**Table 1:** Antitubercular activities of compounds 4(a-f) and 5(b-j) against M.tuberculosis H37Rv

**Compounds**	**MIC (µg/mL)** a **MABA** b
4a	50
4b	50
4c	50
4d	25
4e	50
4f	50
5b	100
5c	50
5d	50
5e	50
5f	50
5g	50
5h	50
5i	25
5j	50
Streptomycin	6.25
Pyrazinamide	3.12
Ciprofloxacin	3.12

a Minimum inhibitory concentration in μg/mL.

b Microplate Alamar Blue Assay (visual).

**Table 2:** Cytotoxicity evaluation of compounds 4d and 5i against THP-1 cell line by MTT assay

**Compound** **s**	**IC**_50_ ** (µg/mL)** a
4d	221.00
5i	210.00

a IC_50_ is the concentration required to inhibit 50% of cell growth and the values are means of three experiments.

*In-vitro assay for evaluation of antimycobacterial activity*

The synthesized compounds were examined for antibacterial activity against *Mycobacterium tuberculosis *H37Rv *in-vitro *employing the Microplate Alamar Blue Assay (MABA) method (36). The antitubercular activity was expressed as the minimum inhibitory concentration (MIC) in µg/mL, in comparison with the standard drugs streptomycin, ciprofloxacin and pyrazinamide. Briefly, sterile water (200 µL) was added to all outer-perimeter wells of 96 well plates to minimize evaporation and maintain humidity. The prepared bacterial suspension (10^8 ^CFU/mL) from a log-phase culture of *Mycobacterium tuberculosis *(20 µL) was added to 180 µL of test compound or drug-containing Middle Brook 7H9 broth in each well so as to make up the volume to 200 µL. A broad range of drug concentrations were checked to precisely determine the MIC, with the entire procedure being repeated a minimum of three times. The plates were then covered and sealed with parafilm and incubated at 37 °C for 5 days. After this period 25 µL of a freshly prepared 1:1 mixture of Alamar blue reagent and 10% tween 80 was added to each well and again incubated for 24 h at 37 °C before being assessed for colour development. A blue colour in the well was interpreted as no bacterial growth, and a pink colour will be scored as growth. The MIC was recorded as the lowest drug concentration that prevents a colour change from blue to pink.

*In-vitro cytotoxicity evaluation*

The cytotoxic effect of the compounds 4d and 5h on THP-1 cells was assessed by MTT assay (37). THP-1 (human acute monocytic leukaemia cell line) cells were cultured in Roswell Park Memorial Institute 1640 medium (RPMI 1640) containing 10% fetal bovine serum. In brief, exponentially growing cells were seeded at 10^4^ cells per well into 96-well plates. After 24 h of incubation time, different concentrations of the test compounds were added to the wells. An equal amount of DMSO was added to the cells used as negative controls. The plates were then incubated for different time intervals (24, 48 and 72 h) at 37 °C in 5% CO_2_ atmosphere, and microscopic examination was carried out and observations were noted every 24 h interval. The cells viability was determined by adding 20 µL of MTT (3-(4, 5-dimethylthiazol-2-yl)-2, 5-diphenyltetrazolium bromide) solution (5 mg/mL in PBS) into each well. The plates were gently shaken and incubated for 3 h at 37 °C in 5% CO_2_ atmosphere. After the supernatant was discarded, 100 µL of DMSO was added to each well in order to dissolve the formazan crystals that had formed due to reduction of MTT by viable cells. The plate was placed on the shaker for 15 min and the optical density was recorded using a microplate reader at a wavelength of 540 nm. The percentage growth inhibition and IC_50_ values were calculated (38). The experiment was performed in triplicate.

## Results and Discussion

*Chemistry*

A general strategy to synthesize substituted fluorobenzimidazoles 4a-f and 5b-j is shown in Figures 2 and 3. The ethers 2 and 3 were prepared from 5-chloro-4-fluoro-2-nitro aniline 1 and phenols via nucleophilic aromatic substitution. Reduction of the nitro group in the ether was effected with stannous chloride dihydrate (SnCl_2_^.^2H_2_O) to yield the unstable *O*-phenylenediamine. A simple protocol was followed for the synthesis of benzimidazole derivatives 4a-f and thioethers 5b-j from the corresponding *O*-phenylenediamines 2a and 3a in moderate to good yields.

The most popular approach for the one-pot synthesis of benzimidazole derivatives involves condensations of ortho phenylenediamines with aldehydes under oxidative conditions, while preparation of substituted-2-mercaptobenzimidazoles can be accomplished by cyclocondensation of substituted ortho phenylenediamines with CS_2_ in an EtOH-KOH solution (39-42). Thioethers can be prepared by* S*-alkylation of substituted-2-mercaptobenzimidazoles using alkyl or aryl alkyl halides. Sodium metabisulphite was employed as a catalyst for the synthesis of benzimidazoles 4a-f in good yields from corresponding diamine 3 and different aldehydes using DMF as solvent. *S*-alkylation of substituted-2-mercaptobenzimidazoles 3b-j by 5-bromo-6-bromomethyl-1,3-benzodioxole 4 was carried out in the presence of powdered potassium carbonate dissolved in dry DMF at room temperature to yield the compounds 5b-j in moderate yields.

All new compounds reported in this research investigation were characterized by spectral data (IR, ^1^H NMR, ^13^C NMR and Mass), and their purity was ascertained by elemental analysis. The presence of NH group in the benzimidazole derivatives was confirmed by the comparative assessment of their IR and ^1^H NMR spectras. In the IR spectra of compounds 4a-f and 5b-j, a broad absorption band was seen at 3370-3523 cm^-1 ^for stretching vibration of NH group. In ^1^H NMR spectra of these compounds singlet detected between *δ* 10.01-15.10 ppm is assignable to NH proton of the benzimidazole ring. The products 5b-j of the reaction between substituted-2-mercaptobenzimidazoles and 5-bromo-6-bromomethyl-1,3-benzodioxole in the ^1^H NMR spectras exhibited singlet between 4.49 and 4.91 ppm accounting for the benzylic methylene protons (2H, -CH_2_S-), further its presence is supported by the IR spectral data with the appearance of the benzylic -CH_2_S- bands in the 2872-2906 cm^-1 ^region. The ^13^C NMR results showed that the compounds with benzylic methylene carbon (-CH_2_S-) appeared between 34.02-39.32 ppm. Analysis of IR,^ 1^H NMR and ^13^C NMR spectral data confirmed the presence of the methylenedioxy group (-OCH_2_O-) in compounds with methylenedioxyphenyl moiety. The presence of this group is distinctly clear from the bands at 2903-2982 cm^-1 ^for aliphatic C-H stretching, in IR spectra. From the ^1^H NMR spectra, methylene protons of methylenedioxy group (-OCH_2_O-) resonated as a singlet between 5.90 and 6.11 ppm, while the ^13^C NMR results showed that the compounds with this functionality presented the expected signals at *δ *101.42-102.12 ppm. The aromatic carbon atoms for the newly synthesized compounds in the ^13^C spectrum were observed at their usual chemical shifts. The characteristic M+2 peak was observed in the mass spectra of the bromo compounds.

*In-vitro antitubercular evaluation*

*In-vitro* antitubercular evaluation results are reported in Table 1. Among the synthesized compounds, the methylenedioxyphenyl substituted fluorobenzimidazole derivatives 4d and 5i exhibited moderate antitubercular activity against *Mycobacterium tuberculosis *H37Rv with MIC values comparable or better than some of the reported naturally occurring methylenedioxyphenyl moiety bearing compounds with antitubercular *in-vitro *activity.

Analog 4a with pyridine ring at 2-position of the benzimidazole ring was found to be less potent than texaline with MIC value of 50 μg/mL, while similar results were recorded among the phenyl counterparts (4b and 4c). Compounds 4a, 4b and 4c were found to be equipotent to sesamin with MIC values of 50 μg/mL, respectively. The unsubstituted methylenedioxyphenyl moiety at C-2 position of the benzimidazole ring led to compound (4d) with improved activity (MIC = 25 μg/mL) as compared to the compounds (4e and 4f) with electron withdrawing –Br and –NO_2_ groups linked to methylenedioxyphenyl moiety (MIC = 50 μg/mL). The unsubstituted fluorobenzimidazole counterpart 5b was synthesized to see its effect on activity profile and was least active against the mycobacterium with MIC value of 100 μg/mL. The compounds 5g and 5h with halo substituents (Cl and F) on the phenyl ring at 4-position exhibited similar antitubercular activity (MIC = 50 μg/mL), in comparison with the compounds (5c-f) with alkyl or aryl substituents. The methylenedioxyphenoxy substituted compound 5i showed good antimycobacterial activity (MIC = 25 μg/mL) as compared to *N*-phenoxyimidazole substituted compound 5j (MIC = 50 μg/mL).

The compounds 4d and 5i showed relatively better activity against *Mycobacterium tuberculosis *H37Rv with MIC values of 25 μg/mL as compared to the antitubercular natural product sesamin (MIC = 50 μg/mL). In addition, the fluorobenzimidazole derivatives 4d and 5i exhibited similar potency to that of texaline with MIC values of 25 μg/mL. Furthermore, their antitubercular activity was comparable to the natural compounds galipinine and graveolinine with MIC values of 16 μg/mL.

*Cytotoxic assay*

The most active compounds 4d and 5i were further examined for their cytotoxic effect on THP-1 cell line using MTT assay. The compounds proved to be nontoxic with IC_50_ values above 200 µg/mL and the results suggest that these compounds exhibited antitubercular activity at non-cytotoxic concentrations. The results recorded in Table 2 showed low toxicity for compounds 4d and 5i towards THP-1 cells.

## Conclusion

New substituted fluorobenzimidazoles were synthesized and their structures were confirmed by spectral data. The fluorobenzimidazole scaffold was explored at the 2- and 6-position with methylenedioxyphenyl moiety. Substituted methylenedioxyphenyl ring was directly linked to the 2-position of the benzimidazole ring by cyclocondensation and via methylene thio linkage by *S*-alkylation keeping in view the SAR details of potent natural anti-TB drugs. Furthermore, synthesis of analogs incorporating methylenedioxyphenyl moiety at the C-6 position of benzimidazole ring was accomplished by nucleophilic substitution reaction of 5-chloro-4-fluoro-2-nitro aniline with sesamol. *In-vitro* antitubercular activity data revealed that the benzimidazole scaffold with methylenedioxyphenyl moiety at C-2 and C-6 were important for antitubercular activity. Thus, our effort targeted towards incorporation of structural motifs presented in natural product lead has led to compounds 4d and 5i with comparable or improved anti-tubercular activity against *Mycobacterium tuberculosis* H37Rv strain in comparison with the previously reported antitubercular natural products. The results for *in-vitro* cytotoxicity evaluation on THP-1 cell line showed compounds 4d and 5i to be significantly less toxic. In conclusion, the promising activity of the two derivatives 4d and 5i suggests their potential as leads for further optimization and development as antitubercular drug candidates for antimycobacterial research.
